# MicroRNA-15a-5p serves as a potential biomarker and regulates the viability and apoptosis of hippocampus neuron in children with temporal lobe epilepsy

**DOI:** 10.1186/s13000-020-00944-w

**Published:** 2020-05-08

**Authors:** Na Li, Jingmei Pan, Wei Liu, Yuanyuan Li, Feng Li, Min Liu

**Affiliations:** 1 Department of Pediatric Medicine, Yidu Central Hospital of Weifang, Weifang, Shandong 262500 P.R. China; 2 Department of Medical Image, Yidu Central Hospital of Weifang, Weifang, Shandong 262500 P.R. China; 3 Department of Emergency Surgery, Weifang People’s Hospital, No. 151, Guangwen Street, Weifang, Shandong 261000 P.R. China; 4 Department of Medical Insurance, Weifang Maternal and Child Health Care Hospital, Weifang, Shandong 261021 P.R. China

**Keywords:** miR-15a-5p, Temporal lobe epilepsy, Hippocampus neuron, Viability, Apoptosis

## Abstract

**Background:**

Temporal lobe epilepsy (TLE) is the most common type of epilepsy, usually starting in childhood. The dysregulation of microRNAs (miRNAs) has been identified in neurological disorders. The current study investigated the expression level and clinical significance of miR-15a-5p in TLE children, and explored its function in regulating cell behaviors of hippocampal neurons.

**Methods:**

The expression level of miR-15a-5p was examined in the serum of 63 TLE children. Primary hippocampal cells were cultured in magnesium-free medium to mimic TLE condition in children. The effect of miR-15a-5p on the viability and apoptosis of hippocampus neuron was assessed using MTT or flow cytometric apoptosis assay.

**Results:**

TLE children had significantly low expression of miR-15a-5p. MiR-15a-5p was of great value for the diagnosis of TLE in children, with high specificity and sensitivity. The expression level of miR-15a-5p was decreased significantly in hippocampal cells treated in the magnesium-free medium. Overexpression of miR-15a-5p attenuated TLE-induced reduction for cell viability, and reversed the cell apoptosis induced by TLE.

**Conclusions:**

MiR-15a-5p is downregulated in children with TLE, and overexpression of miR-15a-5p promoted the viability and inhibited the apoptosis of hippocampal neuron. MiR-15a-5p may be a promising biomarker for the diagnosis of children TLE.

## Introduction

Epilepsy is a common neurological disorder, and is defined as seizures that occur as a result of neuronal discharge in a certain region of the brain [1]. According to statistic, the incidence of epilepsy in children ranges from 33 to 82 per 100,000 children per years [2]. Temporal lobe epilepsy (TLE) is the most common type of focal epilepsy with seizure onset from the temporal lobe structure [3]. Unfortunately, children with TLE are often difficult to control with one medication, and two or more appropriate medications are always demanded [4]. The goal of treatment for epilepsy is to reduce the duration of seizures, which is related to neuroprotection. Neuroprotection is considered to be an important aspect of preventing the development of epilepsy.

MicroRNAs (miRNAs) are small noncoding RNAs, which function to regulate multiple developmental processes at the posttranscriptional level. A series of reports have proved the importance of miRNAs dysregulation in the nervous system, and have brought growing body of evidence on miRNA dysregulation in neurological disorders, such as Parkinson’s disease and Alzheimer’s disease [5, 6]. A number of recently published studies have now profiled miRNA expression in experimental models of status epilepticus, and multiple abnormally expressed miRNAs have been identified [7–9]. A major study has determined that circulating miR-15a-5p is aberrantly expressed in adult epilepsy patients [10]. Additionally, miR-15a-5p is also suggested to be at low expression in patients with Alzheimer’s disease, which is closely related to the development of epilepsy [11, 12]. These evidence demonstrate the crucial role of miR-15a-5p in the pathogenesis of neurological disorders. However, the expression of miR-15a-5p in children with TLE is still unclear, because TLE in children is different from the syndrome observed in adults [4].

Therefore, in the presents study, we investigated the expression and clinical value of miR-15a-5p in children with TLE, and further explored its effect on hippocampal neuron viability and apoptosis in a TLE cell model.

## Materials and methods

### Study population and sample collection

Sixty-three children with TLE were collected with the mean age of 9.81 ± 2.79 years old, who were diagnosed according to their clinical seizure semiology, video-electroencephalography (EEG) monitoring, and high-resolution magnetic resonance imaging (MRI) results. According to EEG results, the patients manifested repetitive spikes and spike-and-wave discharges in the left and/or right temporal lobes. According to the MRI results, the abnormal MRI imaging includes tumors, disorders of neuronal migration and cortical organization, vascular malformations, mesial temporal sclerosis, and neocortical sclerosis attributable to brain injury. Patients who had non-epileptic disorders were excluded from the study, including electrolyte disturbances, metabolic disorders, acute brain disease or trauma, and non-epileptic paroxysmal events mimicking epilepsy. Sixty-seven healthy individuals were recruited as the control group, with the mean age of 10.13 ± 2.46 years old. 5 ml peripheral blood samples were collected from each participant and centrifuged immediately, then the serum samples were stored at − 80 °C for further analysis.

The protocol of this study was approved by the Ethics Committee of Yidu Central Hospital of Weifang, and written informed consent was collected from each participant.

### Primary culture of hippocampal neurons from newborn rats

Wistar rats were purchased from the Shanghai Animal Laboratory Center, and were housed in cages with a constant indoor temperature of 25 °C and had free access to food and water. All animals were treated according to the Guide for the Care and Use of Laboratory Animals of the Institute for Laboratory Animal Research. One male rat was housed with a female rat for the generation of newborn rats. As described in previous research, primary hippocampal cells were collected from P0 newborn rats and cultured [13]. Briefly, three slices of hippocampus were collected from the brain and then were digested with 0.5% Trypsin at 37 °C for 15 min. Then the dissociated single cells were washed twice with Hank’s buffered saline solution. After treatment with DNase I, neurons were placed on glass coverslips with plating medium and cultured in a humidified incubator with 5% CO_2_ at 37 °C. After confirming that the cells are viable and have attached, cells were cultured in Neurobasal medium (Invitrogen; Thermo Fisher Scientific, Inc., Waltham, MA, USA). Once a week, replace one-third of the medium with fresh Neurobasal medium. After incubation for 8 days, primary hippocampal neurons were used for subsequent experiment.

### Establishment of epileptiform discharge model in rat hippocampal neurons

To mimic TLE condition in children, hippocampal neurons from newborn rats were cultured in magnesium-free medium (145 g NaCl, 2.5 g KCl, 10 g HEPES, 2 g CaCl2, 10 g glucose, 0.002 g glycine dissolved in 1000 mL distilled water, pH 7.2) at 37 °C for 3 h, and the hippocampal epileptiform activity was induced [14, 15].

### Cell transfection

MiR-15a-5p mimic and its negative control (mimic NC) were provided by Gene-Pharma (Shanghai, China). Rat primary hippocampal neurons were plated on a 96 well plate, and cell transfection was performed at a confluency of 85–90%. According to the transfection, cells were assigned into four groups: control group (untreated cells), No Mg^2+^ (cells were cultured in a magnesium-free medium), No Mg^2+^ + mimic NC (cells were transfected with miR-15a-5p mimic NC, 24 h post-transfection cells were cultured in a magnesium-free medium at 37 °C for 3 h), No Mg^2+^ + miR-15a-5p mimic (cells were transfected with miR-15a-5p mimic, 24 h post-transfection cells were cultured in a magnesium-free medium at 37 °C for 3 h). Lipofectamine 2000 (Invitrogen, Carlsbad, CA, USA) was used for the transfection according to the manufacturer’s protocols. 24 h post-transfection, cells were cultured in a magnesium-free medium at 37 °C for 3 h to establish TLE model. The control group was treated with nonmagnesium-free medium, which is magnesium-free medium with 1 mM MgCl_2_.

### RNA extraction and quantitative real-time polymerase chain reaction (qRT-PCR)

Total RNA was isolated from both human serum and cultured rat hippocampal neurons by using Trizol reagent (Invitrogen, Carlsbad, CA, USA) based on the manufacturer’s protocol. Reverse transcription was performed using TaqMan miRNA reverse transcription kit (Applied Biosystems, Foster City, CA, USA). qRT-PCR assay was performed to determine the gene expression using a SYBR Premix Ex Taq™ II commercial kit (Takara, Dalian, China) and the Applied Biosystems 7900 Real-Time PCR System (Applied Biosystems, Foster City, CA). The following thermocycling conditions were used for the PCR: initial denaturation at 95 °C for 5 min; 30 cycles of 95 °C for 30 s, 60 °C for 30 s and 72 °C for 20 s; and a final extension at 72 °C for 10 min. The relative gene expression was normalized to that of the internal control U6 according to the comparative delta CT (2^−ΔΔCt^) method. The primers used were as follows: miR-15a-5p forward, 5′-GGGUAGCAGCACAUAAUGG-3′ and reverse, 5′-CTCAACTGGTGTCGTGGA-3′; and U6 forward, 5′-CTCGCTTCGGCAGCACA-3′ and reverse, 5′-AACGCTTCACGAATTTGCGT-3′.

### MTT assay

The cell viability was assessed using MTT assay according to the previous research [16]. 50 μl of MTT (Sigma-Aldrich; Merck, Darmstadt, Germany) was added into each well and incubated for further 3 h, followed by addition of 150 μl of dimethyl sulfoxide (DMSO) (Sigma-Aldrich; Merck). The cell viability was determined by measuring the absorbance at 490 nm using a microplate reader.

### Flow cytometric apoptosis assay

An Annexin V-FITC Apoptosis Detection kit (Keygen Biotechnology) was used to detect cell apoptosis. Cells of each group were harvest, and washed with incubation buffer (10 mmol/L HEPES/NaOH, pH 7.4, 140 mmol/L NaCl, 5 mmol/L CaCl_2_). Subsequently, the cells were re-suspended with PBS and mixed with 5 μl Annexin V-FITC and PI staining solution. After incubation for 10–15 min in the dark, SA-FLOUS was added and incubated in the dark for another 20 min. The apoptotic rates were measured using a FACSCalibur flow cytometer (BD Biosciences).

### Statistical analysis

All the data analyses were performed using SPSS version 18.0 software (SPSS Inc., Chicago, IL) and GraphPad Prism 5.0 software (GraphPad Software, Inc., USA). The data were expressed as mean ± standard deviation (SD). Demographic data were analyzed using an independent-samples t-test or a chi-squared test. Differences between two groups were compared using student’s *t*-test or one-way ANOVA analysis. Receiver operating characteristic (ROC) curve analysis was applied to determine the specificity and sensitivity of miR-15a-5p levels regarding the diagnosis of TLE. Each experiment had at least three repetitions. *P* < 0.05 was considered to be statistically significant.

## Results

### The demographic and clinical characteristics of the study population

The demographic and clinical characteristics of both case and control groups were summarized in Table 1. The age of the individuals ranged from 6 to 14 years old in the healthy controls group, and 5–15 years in the TLE patients group. There was no significant difference for the age and gender distribution between the control and TLE patients group (*P* > 0.05, Table 1).

**Table 1:** Clinical characteristics of the patients with TLE and the healthy controls

Parameters	Healthy controls (*n* = 67)	TLE patients (*n* = 63)	*P*-value
Age (years)	10.13 ± 2.46	9.81 ± 2.79	0.482
Gender			
Male	35	33	0.987
Female	32	30	
Interictal EEG			
Unilateral temporal	–	47	
Bilateral temporal	–	16	
Epilepsy type			
Simple partial	–	–	
Complex partial	–	63	
Brain MRI finding			
No abnormality	–	52	
Mesial sclerosis	–	11	

*TLE* temporal lobe epilepsy, *EEG* electroencephalography, *MRI* magnetic resonance imaging

### MiR-15a-5p expression level in the serum of children with TLE

**Fig. 1:**
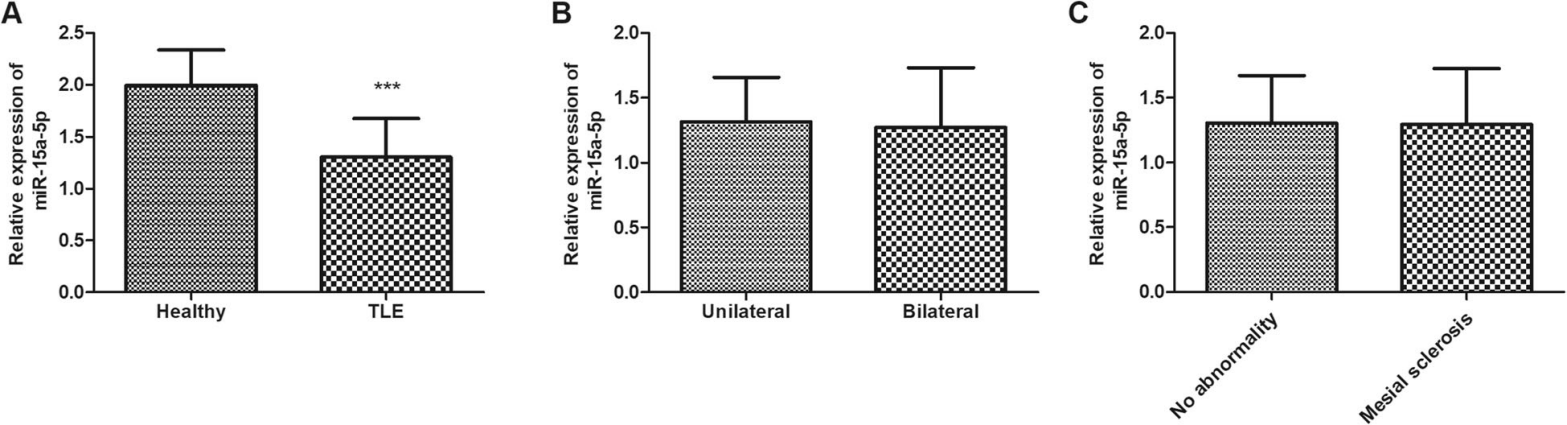
The expression level of miR-15a-5p in the serum of children with temporal lobe epilepsy (TLE) and healthy controls. **a**. The TLE children had significantly low expression of miR-15a-5p compared with healthy group. **b**. There was no significance for miR-15a-5p expression in bilateral temporal patients compared with unilateral temporal patients. **c**. There was no significance for miR-15a-5p expression in mesial sclerosis patients compared with no abnormality patients. *** *P* < 0.001

The expression level of miR-15a-5p in the serum of children with TLE was estimated using qRT-PCR. It was noted that compared with the control group, the TLE children had significantly low expression of miR-15a-5p (*P* < 0.001, Fig. 1), suggesting the potential role of miR-15a-5p in the pathogenesis of TLE. Furthermore, the expression level of miR-15a-5p was compared in the clinical groups of TLE patients according to the results of EEG and MRI. As shown in Fig. 1b, there was no significance for miR-15a-5p expression in bilateral temporal patients compared with unilateral temporal patients (*P* > 0.05). Besides, similar result was also observed in no abnormality and mesial sclerosis groups (*P* > 0.05, Fig. 1c). These data indicated that miR-15a-5p expression did not vary from different subgroups of TLE patients.

### The diagnostic value analysis of serum miR-15a-5p for TLE

**Fig. 2:**
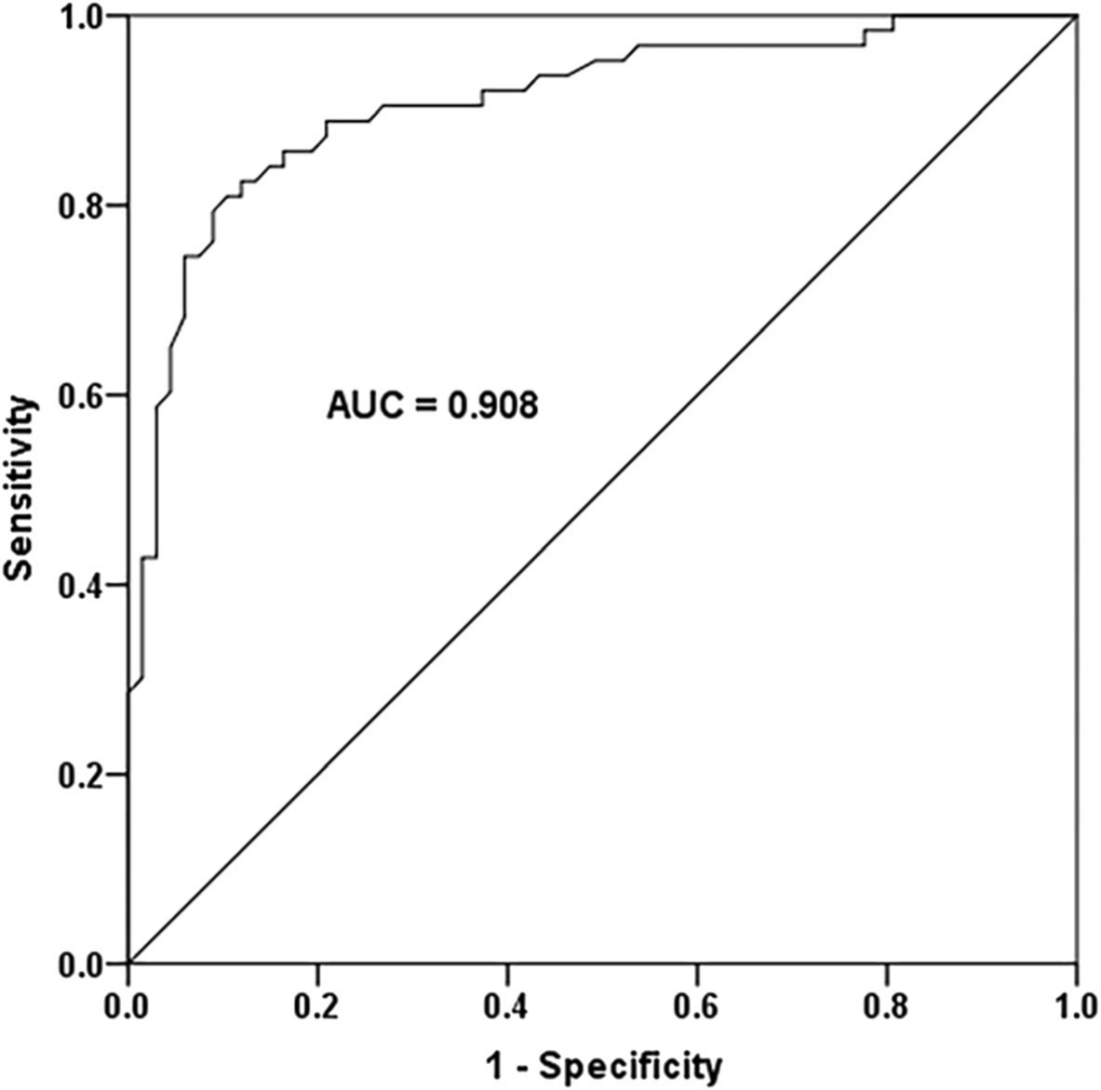
A receiver operating characteristic ROC curve was conducted to calculate the diagnostic value of serum miR-15a-5p for TLE. The area under the curve (AUC) for miR-15a-5p was 0.908, with a sensitivity of 82.5% and specificity of 88.1% at the cutoff value of 1.650

A ROC curve was conducted to calculate the diagnostic value of serum miR-15a-5p for TLE. As shown in Fig. 2, the area under the curve (AUC) for miR-15a-5p was 0.908, with a sensitivity of 82.5% and specificity of 88.1% at the cutoff value of 1.650. These data revealed that miR-15a-5p might be a sensitive biomarker to distinguish TLE patients from healthy individuals.

### Expression level of miR-15a-5p in TLE cell model

**Fig. 3:**
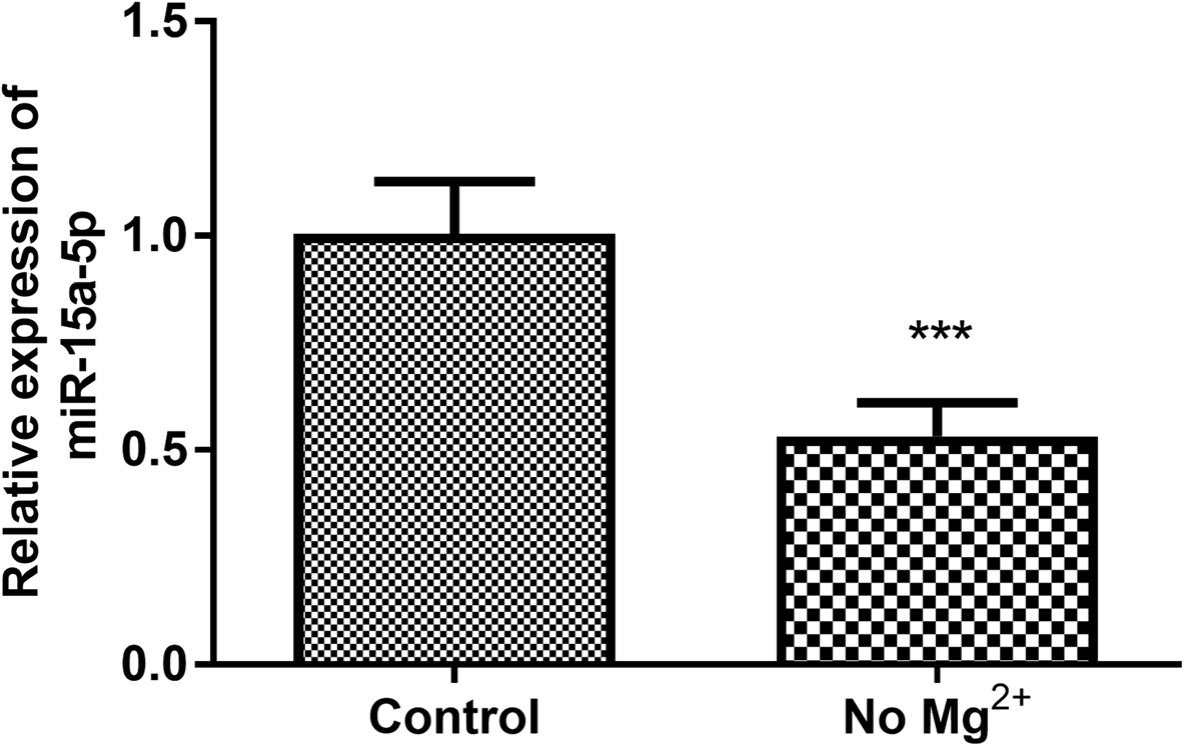
To mimic TLE condition in children, primary hippocampal cells were collected from newborn rats, and cultured in the magnesium-free medium for 3 h to establish TLE cell model. The expression level of miR-15a-5p was decreased significantly in hippocampal cells treated in magnesium-free medium. *** *P* < 0.001

The TLE cell model was established to explore the expression level of miR-15a-5p in vitro. To mimic TLE condition in children, primary hippocampal cells were collected from newborn rats, and cultured in the magnesium-free medium for 3 h. As shown in Fig. 3, compared with the control group, the expression level of miR-15a-5p was decreased significantly in hippocampal cells treated in magnesium-free medium (*P* < 0.001).

### Effects of miR-15a-5p on cell viability and apoptosis

**Fig. 4:**
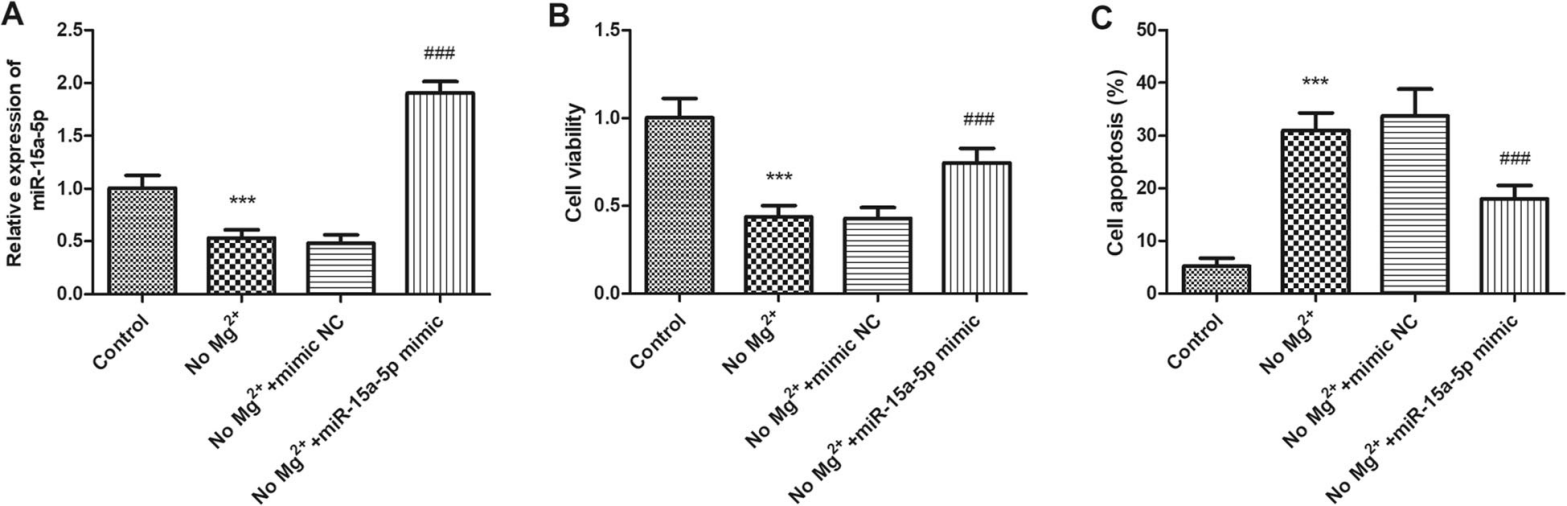
The effect of miR-15a-5p on the viability and apoptosis of hippocampal neuron. **a**. Transfection of miR-15a-5p mimic lead to a remarkable increase in the expression level of miR-15a-5p. **b**. The viability of hippocampal cells treated in the magnesium-free medium was significantly reduced which was reversed by miR-15a-5p mimic transfection. **c**. The cell apoptosis of hippocampal cells treated in the magnesium-free medium was significantly increased, overexpression of miR-15a-5p attenuated TLE-induced cell apoptosis. *** *P* < 0.001, compared with control group; ^###^*P* < 0.001, compared with No Mg^2+^ group

To explore the role of miR-15a-5p in children with TLE, cell transfection was performed to regulate the expression level of miR-15a-5p in hippocampal cells. The overexpression efficiency was examined by using qRT-PCR, and the results suggested that transfection of miR-15a-5p mimic lead to a remarkable increase in the expression level of miR-15a-5p (Fig. 4a, *P* < 0.001). It was found that compared with the control group, the viability of hippocampal cells treated in the magnesium-free medium was significantly reduced, while the cell apoptosis was remarkably increased (*P* < 0.001). As shown in Fig. 4b, transfection with miR-15a-5p mimic significantly attenuated TLE-induced reduction for cell viability (*P* < 0.001). Additionally, overexpression of miR-15a-5p also attenuated TLE-induced cell apoptosis (Fig. 4b, *P* < 0.001).

## Discussion

TLE is one of the most common and intractable forms of epilepsy, which is characterized by recurring seizures due to abnormal neuronal excitability, usually starting in childhood. It has been proved that brain may be at greater risk for epilepsy attack early in life, and the mechanism for the occurrence of epilepsy in immature brain is different from that in the adult brain [17, 18]. As a result of its complex and nonspecific clinical manifestations, TLE is easy to be misdiagnosed or missed [19, 20]. Recently, EEG and neuroimaging have been widely used in the clinical diagnosis of epilepsy which are important auxiliary ways, but the definite diagnosis is primarily according to a detailed examination of clinical manifestations and a thorough medical history. Therefore, biomarkers may contribute to the accurate clinical diagnosis of epilepsy.

As a result of being stabile in serum, economical, rapid and noninvasive, a number of miRNAs have been identified as potential diagnostic tools for many diseases, including neurodegenerative disorders, such as Parkinson’s disease and Alzheimer’s disease [5, 6]. The levels of certain miRNAs have been reported to be altered in the blood of epilepsy patients, such as miR-106b-5p, miR-130a-3p, and miR-146a-5p, and their diagnostic value for epilepsy has also been confirmed [10]. In the present study, the serum expression level of miR-15a-5p was determined to be decreased in children with TLE. Consistently, the dysregulation of miR-15a-5p has been reported in adult epilepsy patients by Wang et al. [10]. Additionally, miR-15a-5p is also reported to be downregulated in patients with Alzheimer’s disease, which shows a remarkable correlation with the occurrence of epilepsy [11, 12]. These data all suggest the important role of miR-15a-5p in the pathogenesis of neurological disorders, supporting our present results.

Accumulated evidence strongly supports the clinical significance of miR-15a-5p in different types of human diseases. For example, Ramzan et al. have reported that miR-15a-5p is differentially expressed in patients with metabolic syndrome (MetS) compared to healthy individuals, and it is identified as a predictive biomarker for MetS [21]. In acute coronary syndrome (ACS) patients, miR-15a-5p is identified to be at low expression level in patients with ST-segment elevated myocardial infarction (STEMI), and has relatively high discriminatory power for STEMI patients [22]. Another study on colorectal cancer has also suggested that miR-15a-5p is upregulated in tumor compared to normal mucosae, and exhibit dramatic diagnostic value [23]. Clinically, the diagnostic value of miR-15a-5p for TLE was also assessed in TLE children. It was noted that miR-15a-5p was of great value for the diagnosis of TLE in children, with high specificity and sensitivity. We concluded that circulating miR-15a-5p may serve as a promising biomarker for the diagnosis of TLE in children. However, correlation of the expression levels of miR-15a-5p with long-term disease of TLE patients was not detected in the current study. In the future, further researches are needed to explore the clinical value of miR-15a-5p for the evolution of TLE.

The present study further explored the effect of miR-15a-5p on the biological function of hippocampal neuron. To mimic TLE condition in children, primary hippocampal cells were collected from newborn rats, and cultured in the magnesium-free medium for 3 h. It was noted that the expression level of miR-15a-5p was decreased significantly in hippocampal cells treated in magnesium-free medium, which is consistent with the results obtained in TLE children. The function of miR-15a-5p in regulating cell behaviors has widely reported. In human hepatocellular carcinoma (HCC), miR-15a-5p is reported to be aberrantly expressed in both HCC cell lines and clinical HCC specimens compared to controls, and overexpression of miR-15a-5p inhibits cell proliferation [24]. Another study by Chen et al. has identified miR-15a-5p to be a new target for osteoarthritis (OA) diagnosis and therapy, and miR-15a-5p may be involved in the pathogenesis of OA through regulating chondrocytes cell progression [25]. It is well established that cell death occurs after epilepsy, but the mechanisms underlying neuronal death remain unclear [26]. In the current study, the effect of miR-15a-5p on the cell viability and apoptosis of hippocampal cells was further investigated, and cell transfection was performed to regulate the expression level of miR-15a-5p in hippocampal cells. The results demonstrated that overexpression of miR-15a-5p significantly enhanced cell viability and inhibited apoptosis, which might be the potential mechanism of the involvement of miR-15a-5p in the progress of children TLE. Although we provided a novel insight on the clinical and functional role of miR-15a-5p in children TLE, further studies are needed to explore the underlying mechanism of the involvement of miR-15a-5p in TLE. Additionally, the current study did not explore the role of miR-15a-5p in different types of TLE, such as neoplastic or non-neoplastic diseases related TLE, which might be interesting and important for future studies.

## Conclusion

Taken together, miR-105a-5p was identified to be downregulated in children with TLE, and overexpression of miR-15a-5p promoted the viability and inhibited the apoptosis of hippocampal cells. The current study provides evidence for miR-15a-5p as a promising biomarker for the diagnosis of children TLE, and the upregulation of miR-15a-5p may have the potential to improve the treatment of TLE.

## Data Availability

All data generated or analyzed during this study are included in this published article.

## Abbreviations

ACS: Acute coronary syndrome
AUC: Area under the curve
DMSO: Dimethyl sulfoxide
HCC: Hepatocellular carcinoma
MetS: Metabolic syndrome
mimic NC: Mimic negative control
miRNAs: microRNAs
MRI: Magnetic resonance imaging
OA: Osteoarthritis
qRT-PCR: Quantitative real-time polymerase chain reaction
ROC: Receiver operating characteristic
SD: Standard deviation
STEMI: ST-segment elevated myocardial infarction
TLE: Temporal lobe epilepsy
